# Flow Characteristics of the Raw Sewage for the Design of Sewage-Source Heat Pump Systems

**DOI:** 10.1155/2014/503624

**Published:** 2014-05-12

**Authors:** Ying Xu, Yuebin Wu, Qiang Sun

**Affiliations:** ^1^School of Energy and Architecture, Harbin University of Commerce, Harbin 150028, China; ^2^School of Municipal & Environmental Engineering, Harbin Institute of Technology, Harbin 150090, China; ^3^State Key Laboratory of Urban Water Resource and Environment, Harbin Institute of Technology, Harbin 150090, China

## Abstract

The flow characteristics of raw sewage directly affect the technical and economic performance of sewage-source heat pump systems. The purpose of this research is to characterize the flow characteristics of sewage by experimental means. A sophisticated and flexible experimental apparatus was designed and constructed. Then the flow characteristics of the raw sewage were studied through laboratorial testing and theoretical analyses. Results indicated that raw sewage could be characterized as a power-law fluid with the rheological exponent *n* being 0.891 and the rheological coefficient *k* being 0.00175. In addition, the frictional loss factor formula in laminar flow for raw sewage was deduced by theoretical analysis of the power-law fluid. Furthermore, an explicit empirical formula for the frictional loss factor in turbulent flow was obtained through curve fitting of the experimental data. Finally, the equivalent viscosity of the raw sewage is defined in order to calculate the Reynolds number in turbulent flow regions; it was found that sewage had two to three times the viscosity of water at the same temperature. These results contributed to appropriate parameters of fluid properties when designing and operating sewage-source heat pump systems.

## 1. Introduction

In China, coal is most commonly used as the main heat source in municipal heating systems. In addition, energy consumption for heating accounts for 13% of annual total energy consumption [1]. As heating systems using coal lead to environmental pollution, nonrenewable energy consumption, and greenhouse gas production, in order to achieve sustainable energy development and improve environment quality, sewage-source heat pump systems are now considered a feasible and sustainable alternative to conventional cooling and heating systems which also have a positive impact on environmental quality.

Sewage-source heat pump systems can be operated at a high coefficient of performance (COP) without releasing air pollution when properly designed and installed. They are also currently in operation in many countries. For example, in a residential area of Korea, a heat pump system using waste water from local saunas, public baths, and buildings has been effectively utilized not only for water heating but also for heating and cooling [2]. At the beginning of 2004, the first application in China of a sewage-source heat pump system for heating and cooling occurred at Miyun wastewater treatment plant in Beijing [3]. In Japan, a simulation study showed that sewage-source heat pumps could reduce energy consumption by 34%, lower the emission of carbon dioxide (CO_2_) by 68%, and control the generation of nitrogen oxides (NO_*x*_) by 75% compared with conventional air-source heat pumps [4].

Sewage-source heat pumps could employ either raw sewage or treated sewage as the heat source. Typically, sewage-source heat pump systems have been placed after the wastewater treatment plant, utilizing the treated outflow water from the plant as the heat source. However, sewage-source heat pump systems could also use untreated water. The advantage of this is that the placement of these heat pump systems would not be restricted to the locations of wastewater treatment plants; rather, they could be distributed throughout an urban region. Despite these advantages, the design of such untreated sewage-source heat pump systems is made difficult as little is known regarding the fluid properties of untreated sewage. In particular, knowledge regarding raw sewage viscosity and shear-strain relationship and frictional loss factor during turbulent flow is required to appropriately design such systems, as they directly affect the technical and economic performance of the system.

Raw sewage is a kind of two-phase fluid with both solid and liquid phases. The solid is composed of various components, for example, the plastic. In general, raw sewage if used as a heat source in a heat pump system would need to be filtered. After filtration by grids with pore size being 3 mm it may be regarded as a kind of single-phase fluid [5]. The flow characteristics of the heat source directly affect the technical and economic performance of heat pump systems. Therefore, the study on flow characteristics of the raw sewage is of a considerable significance to the design and operation of sewage-source heat pump systems.

The purpose of the paper is to identify the rheological parameters and the constitutive equation of raw sewage by the use of laboratorial testing and theoretical analyses. Then the expressions of frictional loss factor in laminar flow and turbulent flow are determined, respectively, and frictional head losses of the raw sewage flowing in pipelines can be calculated conveniently.

## 2. Experimental System 

### 2.1. Design of the Horizontal-Pipe Rheometer

The measurement of rheological parameters is the process of applying a shear stress on a fluid specimen and tracing the relationship between shear strain and time under certain conditions. In order to parameterize the shear-strain constitutive relationship equation, as shown in (1) [6]

(1) $$\begin{array}{c} \tau =f\left(\dot{\gamma }\right) \end{array}$$

in which *τ* is shear stress, Pa, and $\dot{\gamma }$ is shear strain velocity, 1/s.

Rotary rheometers are widely used for rheological measurement for convenience. However, in the reference [7] it was found that rotary rheometers had some disadvantages in precision. In this study, a horizontal-pipe rheometer is used and its theoretical principle is the Hagen-Poiseuille equation [8], being

(2) $$\begin{array}{c} Q=\frac{\pi R^{4}\Delta P}{8\mu L} \end{array}$$

in which *Q* is flow rate, m^3^/s; *R* is pipe radius, m; Δ*P* is pressure drop along the pipe, Pa; *μ* is dynamic viscosity, Pa · s; *L* is pipe length, m.

By substituting the Robinowitsch-Mooney equation [9] into (2), the following relationship can be found as

(3) $$\begin{array}{c} \frac{\Delta PD}{4L}=k^{\prime }{\left(\frac{8v}{D}\right)}^{n^{\prime }} \end{array}$$

in which *D* is diameter of horizontal pipe rheometer, m; *v* is average velocity at cross section, m/s; *k*′ is rheological coefficient of fluid, *k*′ = *μ* for the Newtonian fluid; *n*′ is rheological exponent of fluid, *n*′ = 1 for the Newtonian fluid.

Giving the experimental pipe diameter and length, the pressure drop along the pipe, Δ*P*, and the flow rate, *Q*, could be measured. Given the flow rate at a cross section, the average velocity at cross section could be calculated. As a result, the rheological coefficient, *k*′, and rheological exponent, *n*′, could be obtained through the use of curve fitting.

### 2.2. Apparatus of Experiments

**Figure 1:**
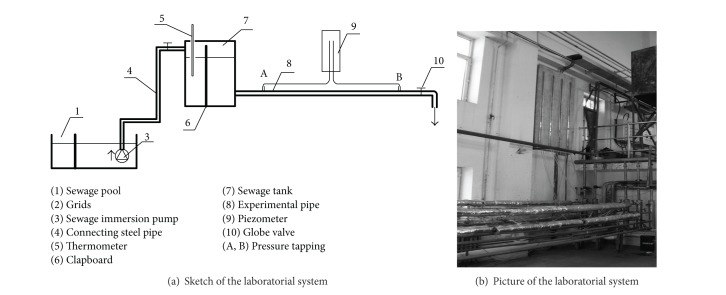
Sketch of the laboratorial system.

The sketch and the picture of the horizontal-pipe rheometer are shown in Figures 1(a) and 1(b), respectively. In order to maintain continuous sewage flow within the rheometer, the experimental system is established in an operating sewage treatment plant, making use of the sewage pool and grids of the treatment plant. The pore size of the grids was 3 mm. Besides the sewage pool and grids, the experimental system consisted of a sewage immersion pump, a segment of connecting steel pipe, a pressure stabilized tank with an overflow pipe, a segment of experimental steel pipe, a globe valve, and a piezometer connected at pressure tapping; also clapboard was used in order to maintain a steady flow in the rheometer.

The rated flow and head of the sewage immersion pump were 25 m^3^/h and 15 m, respectively. The filtrated sewage from the grids was pumped from the sewage pool to the pressure stabilized tank through a steel pipe with diameter of 50 mm. The pressure stabilized tank had dimensions of 1.5 × 1.5 × 1.5 m^3^. The internal diameter and the length of the rheometer pipe were 10 mm and 6.5 m, respectively. The distance from the upstream pressure tapping to the pressure stabilized tank was 1.2 m in order to take into account the entrance effect, which occurs within a distance of 100 to 120 times of the rheometer's diameter [10]. The distance between the two pressure tappings was 5 m. The pressure drop Δ*P* was measured by the piezometer. The flow rate, *Q*, was measured using the volumetric method. Different flow rates were obtained by changing the opening of globe valve.

### 2.3. Calibration of the Horizontal-Pipe Diameter

The diameter of the horizontal pipe was preciously determined in order to calibrate the rheometer. The calibration experiments were performed in laminar flow by the use of a liquid of known viscosity based on the Darcy-Weisbach formula [11] shown as (4). The frictional loss factor under laminar flow conditions satisfies (5) [11]. Therefore, the calibrated diameter can be expressed by (6). Consider the following:

(4) hf=λLDv22g,

(5) λ=64Re,

(6) D4=128υLQπ·g·hf

in which *h*_*f*_ is frictional loss, mH_2_O; *λ* is frictional loss factor; *Re* is Reynolds number; *g* is gravitational acceleration, m/s^2^; *υ* is kinematic viscosity, m^2^/s.

In the study, the calibration of the pipe diameter was performed using the tap water. The results show that the diameter of the experimental pipe was 10.4 mm.

## 3. Identification of Rheological Parameters

After calibrating the horizontal-pipe rheometer, the experimental system was tested at twenty different openings of the valve in order to measure the system response under twenty separate flow conditions.

**Figure 2:**
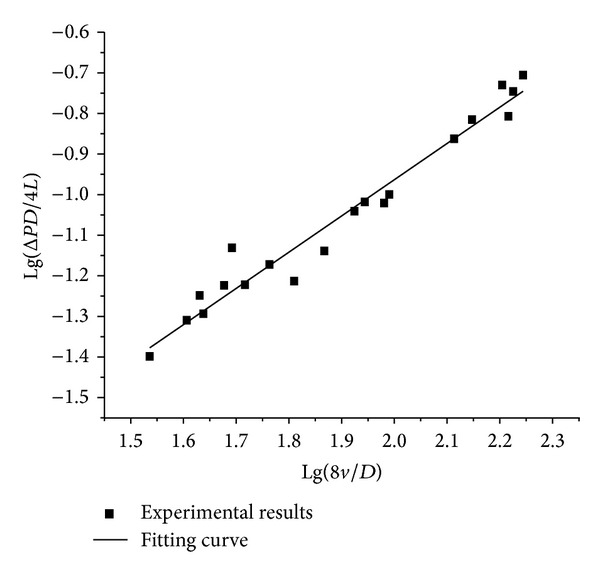
Fitting curve of experimental results.

Figure 2 shows that the fitting curve is linear and does not pass through the origin. Therefore, it can be concluded that the raw sewage exhibits the characteristics of a power-law fluid [12]. The fitting curve also indicates that the rheological coefficient *k*′ was 0.00196 and the rheological exponent *n*′ was 0.891.

A power-law fluid satisfies the following formula [13]

(7) $$\begin{array}{c} \tau =k{\left(\overset{•}{\gamma }\right)}^{n} \end{array}$$

in which *n* is rheological exponent of power-law fluid, *n* = *n*′, *k* is rheological coefficient of power-law fluid, *k* = *k*′/((1+3*n*)/4*n*)^*n*^.

Thus the constitutive equation of the raw sewage could be expressed as follows:

(8) $$\begin{array}{c} \tau =0.00175{\left(\overset{•}{\gamma }\right)}^{0.891}. \end{array}$$

An error analysis of the experimental data was carried out. In the experiments the measurement errors of *D*, *L*, Δ*P*, and *Q* contribute to the synthetic errors and result in the errors of rheological parameters *n* and *k*. From analyses to the experimental data, the relative synthetic error for each group of data was calculated to be no more than 3%.

## 4. The Frictional Loss Factor Formulas

The Reynolds number is the basis of flow characteristic analysis; for a power-law fluid the equation is different from the Newtonian fluid, being [14]

(9) $$\begin{array}{c} \text{R}{\text{e}}^{\prime }=\frac{\rho^{\prime }D^{n}v^{2-n}}{k8^{n-1}} \end{array}$$

in which Re′ is Reynolds number of the power-law fluid and *ρ*′ is the density of the power-law fluid, kg/m^3^.

Therefore, by substituting the experimentally determined values of *n* = 0.891 and *k* = 0.00175 into (9), the Reynolds number of the raw sewage can be determined as

(10) $$\begin{array}{c} \text{R}{\text{e}}_{s}=\frac{\rho_{s}D^{0.891}v^{1.109}}{0.001395} \end{array}$$

in which Re_*s*_ is Reynolds number of the raw sewage and *ρ*_*s*_ is density of the raw sewage, kg/m^3^.

### 4.1. Formula under Laminar Flow Conditions

When a power-law fluid is in laminar flow, the frictional loss factor satisfies the following equation [14]:

(11) $$\begin{array}{c} \lambda_{l}=\frac{64}{\text{R}{\text{e}}^{\prime }} \end{array}$$

in which *λ*_*l*_ is frictional loss factor of power-law fluid under laminar flow conditions. Therefore, by substituting (10) into (11) the frictional loss factor of raw sewage in laminar flow satisfies

(12) $$\begin{array}{c} \lambda_{ls}=\frac{0.0893}{\rho_{s}D^{0.891}v^{1.109}} \end{array}$$

in which *λ*_*ls*_ is frictional loss factor of the raw sewage in laminar flow.

### 4.2. Empirical Formula under Turbulent Flow Conditions

**Figure 3:**
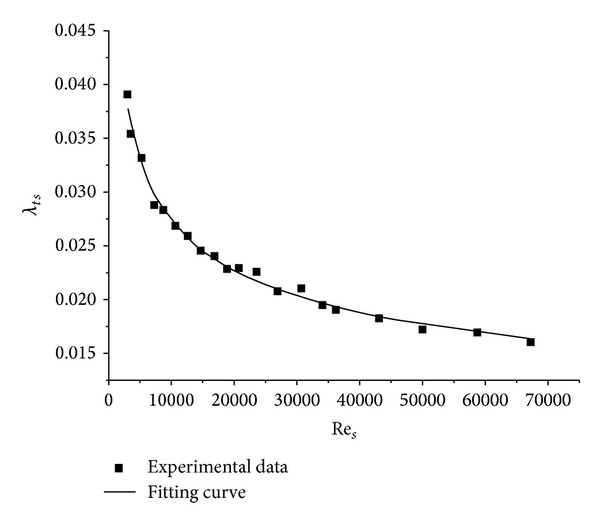
Fitting curve of Re_*s*_ and *λ*_*ts*_.

The same experimental system as in Figure 1 was used to determine the frictional loss factor under hydraulic smooth region. The frictional loss and the flow rate were measured and twenty groups of data were obtained at different flow rates. Assuming that the density of the raw sewage is 1000 kg/m^3^, the frictional loss factor and Reynolds number were calculated according to (4) and (10). The fitting curve is shown in Figure 3 and the empirical formula for the frictional loss factor was found to be

(13) $$\begin{array}{c} \lambda_{ts}=\frac{0.3208}{{\left(\text{R}{\text{e}}_{s}\right)}^{0.27}} \end{array}$$

in which *λ*_*ts*_ is frictional loss factor of the raw sewage in turbulent flow.

The frictional loss factor for a power-law fluid in the hydraulic smooth region under turbulent flow conditions could also be calculated by the Karman formula [15] as follows:

(14) $$\begin{array}{c} \frac{1}{\sqrt{\lambda_{K}/4}}=\frac{4}{{\left(n\right)}^{0.75}}\text{lg}\left\{\text{R}{\text{e}}^{\prime }{\left(\frac{\lambda_{K}}{4}\right)}^{\left[1-(n/2)\right]}\right\}-\frac{0.4}{{\left(n\right)}^{1.2}} \end{array}$$

in which *λ*_*K*_ is frictional loss factor of Karman formula in turbulent flow.

Thus, the Karman formula for raw sewage (*n* = 0.891) could be expressed as

(15) $$\begin{array}{c} \frac{1}{\sqrt{\lambda_{K}/4}}=4.362\cdot \text{lg}\left\{\text{R}{\text{e}}_{s}\cdot {\left(\frac{\lambda_{K}}{4}\right)}^{0.555}\right\}-0.459. \end{array}$$

Comparison between the frictional loss factors calculated by the empirical formula (13) and the Karman formula (15) is shown in Table 1 for the raw sewage in the hydraulic smooth region of turbulent flow.

The results in Table 1 indicate that the differences between *λ*_*ts*_ and *λ*_*K*_ are no more than 10%. Therefore, the presented empirical formula is in good confidence in its capacity to calculate frictional loss factors of the raw sewage in the hydraulic smooth region under turbulent flow conditions.

### 4.3. Equivalent Viscosity

In this study, the equivalent viscosity is defined, by which the Reynolds number of a non-Newtonian fluid is calculated from the Reynolds number expression of a Newtonian fluid under the same turbulent flow conditions. In this way, the Reynolds number of raw sewage in turbulent flow could be expressed as

(16) $$\begin{array}{c} \text{R}{\text{e}}_{s}=\frac{\rho_{s}vd}{\mu_{e}} \end{array}$$

in which *μ*_*e*_ is equivalent viscosity of the raw sewage, Pa · s.

The relationship for *μ*_*e*_ could be obtained by equaling the frictional loss for water with that for raw sewage, as previously determined in this paper.

The frictional loss factor of water can be calculated by Blasius equation [16] as follows:

(17) $$\begin{array}{c} \lambda_{w}=\frac{0.3164}{\text{R}{\text{e}}_{w}^{0.25}} \end{array}$$

in which *λ*_*w*_ is frictional loss factor of water and Re_*w*_ is Reynolds number of water.

Assuming that the frictional loss factor of water *λ*_*w*_ in (17) is equal to that of the raw sewage *λ*_*ts*_ in (13), the relationship between Reynolds number of the raw sewage Re_*s*_ and Reynolds number of water Re_*w*_ satisfies the following equation:

(18) $$\begin{array}{c} \frac{\text{R}{\text{e}}_{s}^{0.27}}{\text{R}{\text{e}}_{w}^{0.25}}=1.0139. \end{array}$$

In engineering practice of sewage-source heat pump systems, the sewage is generally in the turbulent flow and the density *ρ*_*s*_ is regarded as 1000 kg/m^3^, the same as the water density. With the same diameter and flow rate, the ratio of the equivalent viscosity *μ*_*e*_ to the water viscosity can be found as

(19) $$\begin{array}{c} \frac{\mu_{e}}{\mu_{w}}=2\sim 3 \end{array}$$

in which *μ*_*w*_ is viscosity of water, Pa · s.

The results of (19) are detailed in Table 2. Therefore, the equivalent viscosity of the raw sewage is two to three times the viscosity of water at the same temperature. It facilitates the Reynolds number calculation for the raw sewage in engineering practice.

## 5. Conclusions

A sophisticated and flexible experimental apparatus for investigating flow characteristics of raw sewage has been designed and constructed. The rheological parameters and constitutive equation of the raw sewage were determined through laboratorial testing and theoretical analyses, thereby deriving empirical and theoretical frictional loss factor formulas for raw sewage. Consequently, the following conclusions have been drawn.

(1) Raw sewage displays the characteristics of a power-law fluid with rheological exponent, *n*, being 0.891 and the rheological coefficient, *k*, being 0.00175. The constitutive equation is $\tau =0.00175{\left(\overset{•}{\gamma }\right)}^{0.891}$.

(2) The frictional loss factor formula in laminar flow for raw sewage was deduced by theoretical analysis of a power-law fluid and is given by *λ*_*ls*_ = 0.0893/(*ρ*_*s*_ *D* ^0.891^ *v* ^1.109^).

(3) By curve fitting of the experimental data, an empirical formula of the frictional loss factor in turbulent flow for raw sewage was determined as *λ*_*ts*_ = 0.3208/(Re_*s*_)^0.27^. The explicit expression distinguishes itself in simplicity and convenience in comparison with the Karman formula.

(4) The equivalent viscosity of raw sewage was defined, thereby introducing a convenient method for calculating the frictional loss factor of turbulent flow. The ratio of the equivalent viscosity of sewage ranged from two to three times that of water at the same temperature.

The study results contribute to the engineering design and operation of the sewage-source heat pump systems.

## Figures and Tables

**Table 1:** Comparison of *λ*_*ts*_ and λ_*K*_.

Re_*s*_	*λ*_*ts*_	λ_*K*_	Differences
2991.3548	0.03788	0.04093	0.0744
3510.366	0.03630	0.03894	0.0678
5196.7925	0.03269	0.03458	0.0547
7279.2964	0.02988	0.03138	0.0479
8742.9997	0.02845	0.02981	0.0456
10645.961	0.02699	0.02824	0.0442
12646.107	0.02578	0.02698	0.0445
14729.198	0.02475	0.02591	0.0447
16838.074	0.02388	0.02503	0.0458
18992.198	0.02313	0.02428	0.0475
20787.17	0.02258	0.02372	0.0482
23585.573	0.02183	0.022996	0.0508
26976.342	0.02106	0.02224	0.0531
30773.603	0.02033	0.02155	0.0566
34125.68	0.01978	0.02101	0.0587
36318.57	0.01945	0.02071	0.0608
43105.47	0.01858	0.01989	0.0659
50137.31	0.01785	0.01921	0.0708
58836.33	0.01710	0.01852	0.0767
67352.30	0.01649	0.01797	0.0824

Note: *λ*_*ts*_: frictional loss factor of the raw sewage in turbulent flow; *λ*_*K*_: frictional loss factor of Karman formula in turbulent flow.

**Table 2:** Ratio of the equivalent viscosity to the water viscosity.

*λ*_*ts*_	*Re*_*s*_	*Re*_*w*_	*μ*_*e*_/*μ*_*w*_
0.03788	2991.3548	4865.783	1.627
0.03630	3510.366	5772.476	1.644
0.03269	5196.7925	8776.698	1.689
0.02988	7279.2964	12578.743	1.728
0.02845	8742.9997	15297.454	1.750
0.02699	10645.961	18878.141	1.773
0.02578	12646.107	22689.018	1.794
0.02475	14729.198	26701.832	1.813
0.02388	16838.074	30803.925	1.829
0.02313	18992.198	35030.321	1.844
0.02258	20787.17	38577.246	1.856
0.02183	23585.573	44148.115	1.872
0.02106	26976.342	50958.388	1.889
0.02033	30773.603	58654.352	1.906
0.01978	34125.68	65502.310	1.919
0.01945	36318.57	70007.290	1.928
0.01858	43105.47	84063.285	1.950
0.01785	50137.31	98786.534	1.970
0.01710	58836.33	117194.504	1.992
0.01649	67352.30	135396.103	2.010
0.01484	100000	206502.717	2.065
0.009658	500000	1151929.060	2.304
0.008026	1000000	2415048.015	2.415
0.004340	10000000	28243971.830	2.824
